# *Clerodendranthus spicatus* [*Orthosiphon aristatus* (Blume) Miq*.*] maintains uric acid homeostasis via regulating gut microbiota and restrains renal inflammation in hyperuricemic nephropathy

**DOI:** 10.3389/fphar.2024.1485861

**Published:** 2024-11-25

**Authors:** Yang Wang, Kaiwen Li, Siya Yan, Ge Li, Meifang Cheng, Qian Chen, Yuzheng Wu, Dan Wang, Tao Wang

**Affiliations:** ^1^ State Key Laboratory of Chinese Medicine Modernization, Tianjin University of Traditional Chinese Medicine, Tianjin, China; ^2^ Tianjin State Key Laboratory of Modern Chinese Medicine, Tianjin University of Traditional Chinese Medicine, Tianjin, China; ^3^ State key Laboratory of Component-Based Chinese Medicine, Tianjin University of Traditional Chinese Medicine, Tianjin, China; ^4^ State Key Laboratory of Bioactive Substance and Function of Natural Medicines, Institute of Materia Medica, Chinese Academy of Medical Sciences and Peking Union Medical College, Beijing, China

**Keywords:** *Orthosiphon aristatus* (Blume) Miq., hyperuricemic nephropathy, gut microbiota, renal fibrosis, NLRP3 signaling pathway

## Abstract

**Introduction:**

The kidney damage caused by the deposition of uric acid in the kidneys is of urgent need for new treatment drugs due to its complex pathogenesis. *Orthosiphon aristatus* (Blume) Miq. Also known as *C. spicatus*, which has a significant therapeutic effect on hyperuricemia nephropathy (HN), however, the specific mechanism of its action is still unknown.

**Methods:**

The HN mice model was constructed using adenine (AD) and potassium oxonate (PO), and serum biochemical indexes, kidney pathological changes, xanthine oxidase (XOD) activity in the liver, and renal protein expressions of phosphoribose pyrophosphate synthetase (PRPS) and uric acid transporter were detected. The effects of *C. spicatus* on uric acid lowering, anti-inflammation, and renal protection of HN mice were verified. The effect of *C. spicatus* on gut microbiota was assessed by 16 S rRNA sequencing. Establish pseudo-sterile mice through the combined treatment of ampicillin, neomycin, and vancomycin to verify the role of gut microbiota in improving HN in *C. spicatus*.

**Results:**

In HN mice, *C. spicatus* could significantly reduce serum uric acid levels and improve renal function. In addition, *C. spicatus* modulated gut microbiota and decreased the relative abundance of *Bacteroides, Prevotellaceae_UCG-001* and *Alistipes, and increased the abundance of Alloprevotella and Lachnospiraceae_NK4A136_group.C.spicatus* altered the expression of the renal urate transporter and key enzymes in hepatic urate synthesis, leading to a decrease in serum uric acid levels. *C. spicatus* alleviated kidney inflammation by inhibiting the activation of the NLRP3 and TLR4/MYD88 inflammatory pathways, and reduced the level of kidney inflammatory factors. It also improved kidney damage by inhibiting the process of renal epithelial-mesenchymal transition, and improved kidney fibrosis. In pseudo-sterile HN mice, without the effect of gut microbiota, the uric acid lowering, anti-inflammatory, and renal fibrosis improving effects of *C. spicatus* were significantly reduced.

**Conclusion:**

Our results demonstrated that *C. spicatus* could reduce uric acid levels, anti-inflammatory effects, and improve HN by regulating the gut microbiota. This provides a novel scientific basis for the clinical application of *C. spicatus*.

## Introduction

Uric acid, the final product of purine metabolism, maintains dynamic balance in the human body. When this balance is disrupted and blood uric acid rises abnormally, it leads to a metabolic disease called hyperuricemia (HUA) (Zhang et al., 2024). HUA is an independent risk factor for various diseases like chronic kidney disease (CKD), cardiovascular and cerebrovascular diseases, hypertension, and metabolic diseases (Wang et al., 2023). Hyperuricemia is an independent risk factor for kidney disease (Oh et al., 2019). Epidemiological studies show the prevalence of CKD in patients with hyperuricemia (32.7%) is much higher than in those with normal uric acid (16.2%) (Huang et al., 2020). Clinically, kidney diseases caused by HUA such as kidney stones, obstruction, renal interstitial inflammation, acute or chronic renal failure are called hyperuricemic nephropathy (HN), which can be divided into chronic and acute types. Chronic HN has a slow course and insidious onset (Xiao et al., 2019). In the early stage, patients only have mild back pain and mild proteinuria. In the late stage, uric acid crystals block renal tubules, causing renal colic, hematuria, and secondary infection, presenting with symptoms such as urgency, painful urination, and fever. In the late stage, uremia can be induced. Acute HN has a rapid onset (Amano et al., 2024). A large amount of urate microcrystals are rapidly deposited in the renal tubules, collecting ducts, and renal pelvis (Lai et al., 2023). The renal tubule lumen is filled and blocked, resulting in increased pressure in the renal tubules and glomerular sacs, leading to a sharp decline in the glomerular filtration rate and potentially developing into acute renal failure (Su et al., 2020).

Currently, uric acid-lowering therapy (ULT) is the mainstay treatment for HN (Liu et al., 2021). Clinical medication of HN patients requires considering both lowering uric acid and regulating renal function effects of drugs (Wen et al., 2024). However, whether these drugs possess renal protective effects warrants further research. For instance, allopurinol can significantly inhibit xanthine oxidase (XOD) activity and reduce uric acid levels in patients, but is unsuitable for long-term use in patients with impaired renal function (Stamp and Chapman, 2020); benzbromarone, a first-line medication for promoting uric acid excretion, has adverse reactions like liver and kidney side effects. Current uric acid-lowering drugs have significant limitations for HN patients with renal dysfunction (Wu et al., 2024). Therefore, finding drugs with fewer adverse reactions, significant efficacy, and suitability for long-term use in HN patients is urgent.

*Orthosiphon aristatus* (Blume) Miq. (Zhu et al., 2023), also known as *C. spicatus* (Thunb.) C.Y.Wu is mainly distributed in Fujian, Hainan, and other places in China (Zhang et al., 2023). According to the records in the Dai medical books “Ye Bei Jing” and “Fang Ha Ya”, *C. spicatus* has a medicinal history of more than 2000 years in China, and can treat urinary system diseases such as dysuria, fever, and pain (Li et al., 2022). *C. spicatus* contains rich chemical components, including diterpenoids, triterpenoids, flavonoids, phenols, and phenolic acids (Dias et al., 2021). It has functions such as diuresis and stone excretion, uric acid lowering, anti-inflammatory, antioxidant, antibacterial, hypoglycemic, antihypertensive, and immune regulation (Li et al., 2024). *C. spicatus* ethanol extract and total flavonoids extract can reduce the uric acid level of HUA mice. The water extract of *C. spicatus* reduces the level of oxidative stress in rats with chronic renal failure, inhibits the expression of Fibroblast growth factor (b-FGF) in renal tubules and renal interstitium, and improves renal function (Zhou et al., 2024). The multi-methoxy group flavonoid in *C. spicatus* has also been proved to promote sodium excretion and diuresis, and treat kidney stones.

As an important site for oral drug metabolism, gut microbiota has a biotransformation effect on the chemical components of traditional Chinese medicine, promoting changes in drug chemical structure and yielding more biologically active substances for therapeutic effect (Zimmermann et al., 2019). Traditional Chinese medicine could regulate intestinal balance by structuring intestinal microbial flora, affecting host health (Wang et al., 2024). Recent evidence suggests that dysbiosis of gut microbiota plays a crucial role in the pathogenesis of HUA. Altered microbiota compositions, with reduced *Coprococcus* abundance, were observed in HUA subjects compared to normouricemia subjects. Similarly, *Prevotella*, *Dehalobacterium*, *Ruminococcus*, and *Lactobacillus* levels were notably lower in a rat HUA model (Wei et al., 2022). However, the mouse model was inadequate in fully replicating human uric acid metabolism and could not capture the cross-sectional properties evident in human studies (Wang et al., 2022). As such, while research has indicated that gut microbiota may represent a promising therapeutic target for hyperuricemia treatment, the precise species involved and their specific roles remain uncertain (Álvarez-Lario and Macarrón-Vicente, 2010).

The research findings reveal that the dynamic changes in gut microbiota are deeply associated with the pathogenesis of HN, and intervention strategies targeting these microorganisms may open up new pathways for HN treatment. Based on this, we propose the hypothesis that *C. spicatus* may exert therapeutic potential on HN by regulating the structure of intestinal microbiota. Therefore, we designed and implemented a series of experiments focusing on the therapeutic effects of *C. spicatus* on HN mouse models. By introducing a pseudo sterile mouse model and conducting a detailed analysis of the gut microbiota, we have delved into the possible mechanisms of kidney tea intervention in HN, providing a novel perspective and scientific basis for the treatment of HN.

## Materials and methods

### Materials

The stems of *C. spicatus* were collected from Yunnan, China and identified by Dr. Tao Wang. The HPLC analysis of *C. spicatus* were shown in Supplementary Figure S1, and the relative content of sinensetin in C. spicatus was 0.073%. A voucher specimen was deposited at the Institute of Traditional Chinese Medicine, Tianjin University of TCM. Extracts of *C. spicatus* were provided by Tianjin Key Laboratory of Chemistry and Analysis of Traditional Chinese Medicine.

### Animals

This study was performed on specific pathogen-free (SPF)-grade C57BL/6J mice (8–10 weeks old, male). All mice were purchased from Beijing Vital River Laboratory Animal Technology Co., Ltd. Mice were fed a standard diet, with an indoor temperature of 22 C ± 2 C and a fixed artificial light time of 12 h. Before the formal experiment, mice were adaptively fed for at least 7 days. The animal experiment of this study was approved by Science and Technological Committee and the Animal Use and Care Committee of TJUTCM (No. 202108016).

### AD and PO induced HN mice

HN mice model was established by oral administration of potassium oxonate (PO) and AD (Sigma-Aldrich Co., MO, United States). Benzbromarone and ethanol extract of *C. spicatus* were suspended in ultrapure water and orally administrated, ensuring that these were prepared freshly immediately before use. The mice were intragastric administration of adenine (75 mg/kg/day) and PO (200 mg/kg/day). The *C. spicatus* ethanol extract was administered to the low, medium, and high dose groups by gavage at dosages of 100, 200, and 400 mg/kg, respectively. The positive control group received 50 mg/kg benzbromarone by gavage. Both the normal and HN group were administered the corresponding volume of ultrapure water by gavage. One hour later, all groups except the normal group were given 75 mg/kg adenine and 200 mg/kg PO water by gavage. The overall experiment lasted for a total of 25 days. The mice were anesthetized with isoflurane. The mice were then euthanized with carbon dioxide. The carbon dioxide filling rate is 10%–30% of the chamber volume.

Ultra Performance Liquid Chromatography (UPLC) was used to determine serum uric acid levels in mice (Wen et al., 2020). The clearance of uric acid (Cur) and creatinine (Ccr) were then calculated (Liu et al., 2021).

### Antibiotics destroy the gut microbiota of mice

Male C57BL/6J mice were randomly divided into four groups: Blank group treated with antibiotics (Nor-AT), HN group treated with antibiotics (Mod-AT), *C. spicatus* ethanol extract 400 mg/kg group treated with antibiotics (CS400-AT), and *C. spicatus* ethanol extract 400 mg/kg (CS400), with 10 mice per group. Antibiotics (ampicillin 1 g/L, neomycin 1 g/L, vancomycin 500 mg/L) were dissolved in ultra-pure water for mice in the antibiotic groups to drink *ad libitum* during the experiment. Mice in the 400 mg/kg *C spicatus* ethanol extract group received ultra-pure water *ad libitum*.

### Histopathology of renal tissues

Kidney tissue was fixed with 10% formalin and embedded in paraffin. The sections were stained with hematoxylin and eosin (H&E) or Masson’s trichrome (MASSON). The stained sections were observed using an optical microscope at × 400 magnification. The histopathological changes were evaluated by assessing epithelial cell necrosis and renal tubule dilatation. The positively stained area of renal interstitial fibrosis was quantitatively measured using ImageJ software (National Institutes of Health, Bethesda, Maryland, Uinted States).

### Immunohistochemistry

The kidney-embedded wax block was cut into 4 μm slices. After deparaffinization, they were treated with citrate buffer and then 0.3% H_2_O_2_. 3% BSA was used to block non-specific proteins. After blocking, each slide was incubated with CD68 primary antibody (Abcam plc. Cambridge, MA, United States) at 4 C overnight. The next day, the tissue section was incubated with the secondary antibody. Images were observed and captured with an Axio Imager 2. c (Zeiss, Oberkochen, Germany).

### Western blot

The methods for Western blotting were consistent with our previous studies (Wang et al., 2023). Antibodies used in this study included: anti-PRPS (Abcam plc. Cambridge, MA, United States), anti-URAT1(ProteinTech Group. Chicago, United States), anti-GLUT9 (Millipore Co. Ltd. Bedford, MA, United States), anti-ABCG2 (Abcam plc. Cambridge, MA, United States), anti-OAT1 (Abcam plc. Cambridge, MA, United States), anti-IL-1β (Abcam plc. Cambridge, MA, United States), anti-IL-18 (Abcam plc. Cambridge, MA, United States), anti-IL-6 (Abcam plc. Cambridge, MA, United States), anti-MCP-1 (Abcam plc. Cambridge, MA, United States), anti-TNF-α (Abcam plc. Cambridge, MA, United States), anti-E-cadhrein (Abcam plc. Cambridge, MA, United States), anti-α-SMA (Abcam plc. Cambridge, MA, United States), anti-NLRP3 (Abcam plc. Cambridge, MA, United States), anti-pro-Caspase1 (Abcam plc. Cambridge, MA, United States), anti-ASC (Abcam plc. Cambridge, MA, United States), anti-ERK (Abcam plc. Cambridge, MA, United States), anti-phosphor-ERK (Abcam plc. Cambridge, MA, United States), anti-IκKα anti-IκKβ(Abcam plc. Cambridge, MA, United States) (Abcam plc. Cambridge, MA, United States), anti-IκKβ (Abcam plc. Cambridge, MA, United States), anti-MYD88 (Abcam plc. Cambridge, MA, United States), anti-NF-κB (Abcam plc. Cambridge, MA, United States) and β-actin (Abcam plc. Cambridge, MA, United States) antibodies.

### Collection of mouse feces and detection of intestinal bacteria 16 S rDNA gene

We collected fecal samples from mice and analyzed them for the presence of intestinal bacteria using PCR amplification of the 16 S rDNA gene. This gene is commonly used as a marker for bacterial identification and has been shown to be present in a wide variety of bacterial species. To prepare the samples for PCR, we first homogenized the feces in sterile water using a mortar and pestle. We then centrifuged the homogenate at 10,000 rpm for 10 min to pellet any remaining solids. The supernatant was collected and used as the template for PCR amplification of the 16 S rDNA gene. The resulting PCR products were visualized on a 1% agarose gel to confirm amplification of the 16 S rDNA gene.

### Statistical analysis

The result data are shown as the mean ± S.E.M. The SPSS 20.0 statistical software was used for data analysis (version 20, SPSS; IBM, Armonk, NY, United States). The significant differences between the data were evaluated by one-way ANOVA, LSD and Dunnett’s (Tukey) test were used for *post hoc* evaluations. *p* < 0.05 was considered statistically significant.

## Results

### *C. spicatus* lowered uricemia and attenuated renal injury in HN mice

**FIGURE 1:**
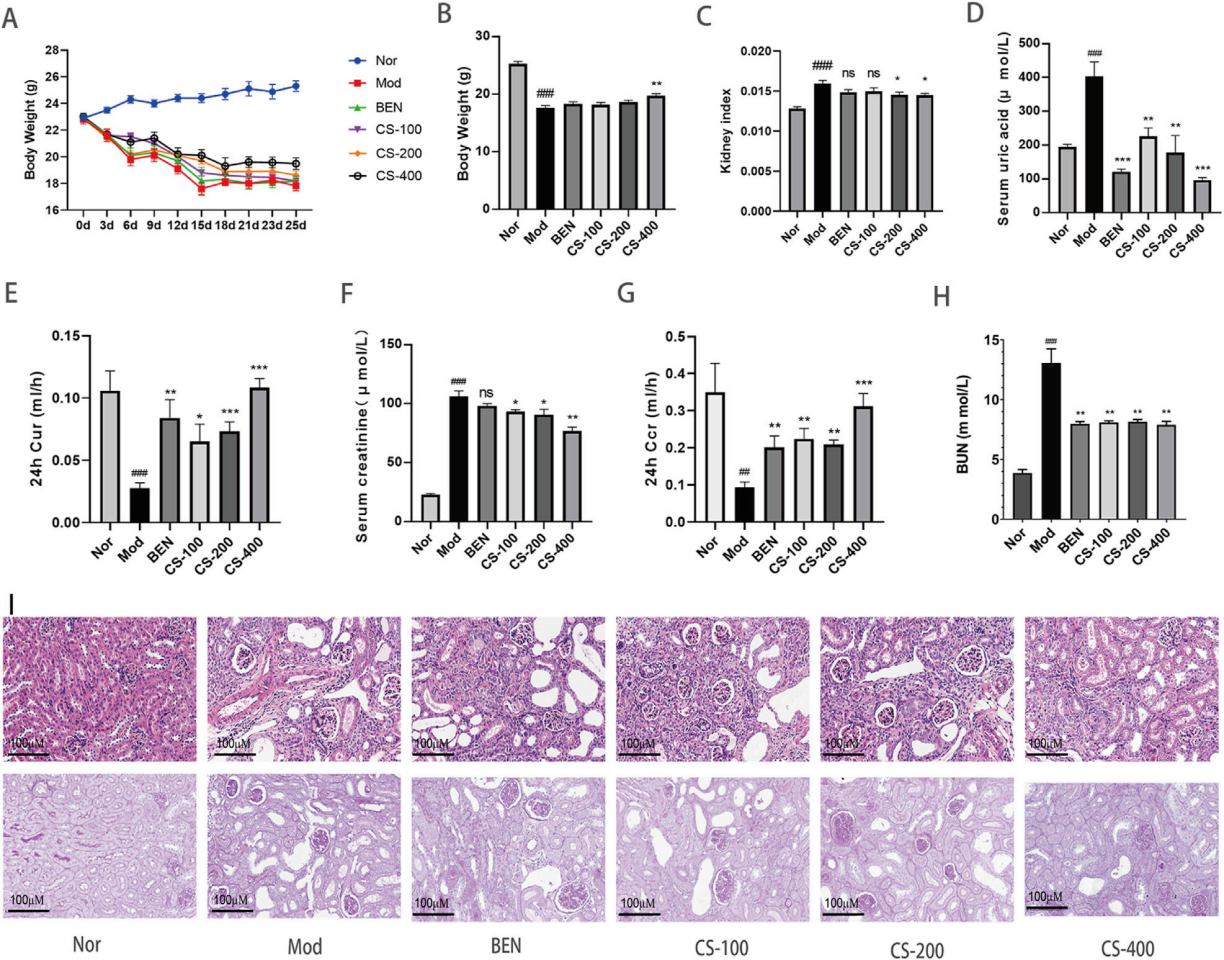
*C. spicatus* lowered uricemia and attenuated renal injury in HN mice **(A)** Body weight; **(B)** Body weight; **(C)** Kidney index; **(D)** Serum uric acid; **(E)** 24 h Cur; **(F)** Serum creatinine **(G)** 24 h Ccr; **(H)** BUN; **(I)** H&E and PAS (200 ×) staining in mice kidneys. Nor: blank control group; Mod: hyperuricemia nephropathy group; BEN: benzbromarone 50 mg/kg; CS-100: Extracts of *C. spicatus*, low dose group 100 mg/kg; CS-200: Extracts of *C. spicatus*, medium dose group 200 mg/kg; CS-400: Extracts of *C. spicatus*, high dose group 400 mg/kg. Data are expressed as mean ± SEM (n = 10). Compared with Nor group, ^#^ *p* < 0.05, ^##^ *p* < 0.01, ^###^ *p* < 0.001; Compared with HN group, *p < 0.05, **p < 0.01, ***p < 0.001.

During the experiment, the HN mice exhibited health decline, slow response, emaciation, and withered fur (Figures 1A, B). Compared to the normal group, the kidney coefficient in the HN group of mice significantly increased (*p* < 0.001), indicating that the combination of PO and AD successfully induced renal injury. Compared to the HN group, the kidney coefficient of mice improved after treatment with medium and high doses of *C. spicatus* ethanol extract, indicating that *C. spicatus* ethanol extract has a certain renal protective effect on mice with HN (Figure 1C). Compared to the normal group, the HN group of mice showed significantly elevated serum uric acid levels and reduced 24 h uric acid clearance rate (*p* < 0.001), indicating successful model establishment. Renal dysfunction impairs uric acid excretion, resulting in elevated levels. In comparison to the HN group, treatment with BEN and low, medium, and high doses of *C. spicatus* ethanol extract resulted in varying degrees of reductions in serum uric acid concentrations and increases in the 24 h uric acid clearance rate. These findings suggest that BEN and *C. spicatus* ethanol extract could promote renal uric acid excretion to varying extents, thereby lowering serum uric acid levels in mice with the HN (Figures 1D, E). Figure 1D clearly demonstrates that *C. spicatus* ethanol extract dose-dependently reduces serum uric acid levels compared to the HN group. This pronounced dose-response strongly supports the potential efficacy of CS in managing hyperuricemia. Compared to the normal group, the HN group exhibited significantly increased serum creatinine and BUN levels (*p* < 0.001), and decreased 24 h creatinine clearance. These significant changes in three renal function injury indicators suggest impaired renal function in HN mice, successfully establishing the HN model. Compared to the HN group, BEN treatment did not significantly change serum creatinine, but significantly reduced BUN, indicating some renal protective effects. After treatment with low, medium, and high doses of *C. spicatus* ethanol extract, there was a significant decrease in both serum creatinine and BUN, and increase in 24 h creatinine clearance, indicating improved renal function injury (Figures 1F–H). The H&E results showed that compared to the normal group, the mice in the HN group exhibited renal tubular lumen dilation, inflammatory infiltration, and renal tubular epithelial cell necrosis in the kidney tissue, indicating significant structural damage to the kidneys. In contrast, the mice in the BEN group showed improved renal tubular lumen dilation and more intact and clear boundaries of the renal tubules compared to the HN group. After administration of high, medium, and low doses of *C. spicatus* ethanol extract, significant improvements in renal tubular lumen dilation, inflammatory infiltration, and renal tubular epithelial cell necrosis were observed, with clear and intact boundaries of the renal tubules. The results revealed that *C. spicatus* ethanol extract significantly improved the renal structural damage in HN mice, and the high-dose group of *C. spicatus* ethanol extract showed better reversal of kidney damage compared to the BEN group. The PAS results indicate that, compared to the normal group, HN group exhibited renal cell infection and thickened cell membranes. In contrast, the mice treated with BEN receiving low, medium, and high doses of *C. spicatus* ethanol extract showed improvements in renal cell infection and membrane structure thickening and hyperplasia. The results demonstrate that *C. spicatus* ethanol extract significantly inhibits renal inflammation in HN mice and improves kidney damage (Figure 1I).

### *C.spicatus* reduced the uric acid level by regulating the expression of uric acid transporter and urate synthesis key enzymes in HN mice

**FIGURE 2:**
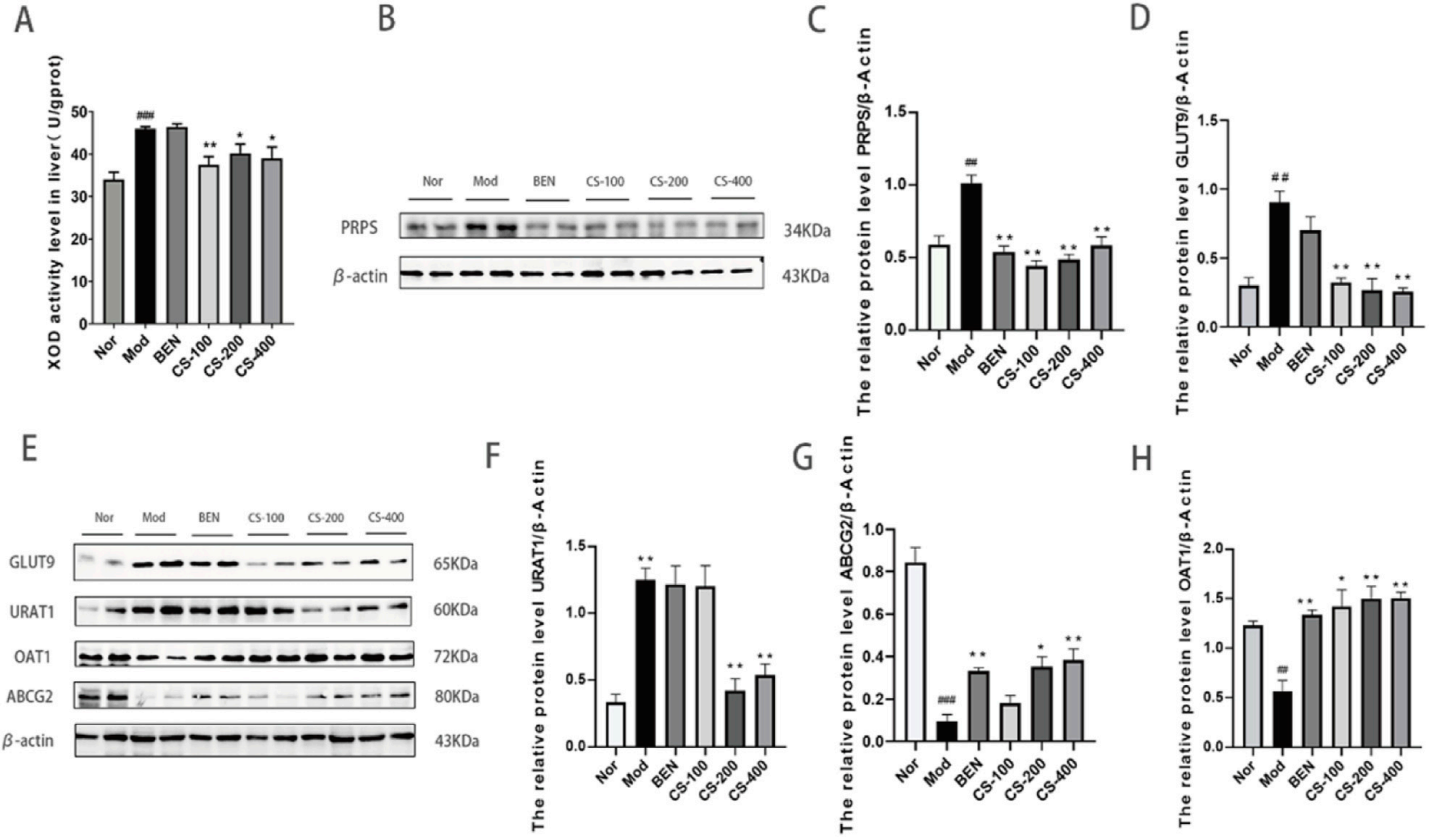
*C. spicatus* reduced the uric acid level by regulating the expression of uric acid transporter and urate synthesis key enzymes in HN mice **(A)** XOD activity level in liver; **(B)** *C. spicatus* reduced the expression of PRPS; **(C)** The ratio of PRPS to β-actin was calculated; **(D)** The relative protein level GLUT9 to β-actin; **(E)** *C. spicatus* regulated the expression level of URAT1, ABCG2 and OAT1; **(F–H)** The ratio of URAT1, ABCG2 and OAT1 to β-actin was calculated. Nor: blank control group; Mod: hyperuricemia nephropathy group; BEN: benzbromarone 50 mg/kg; CS-100: Extracts of *C. spicatus*, low dose group 100 mg/kg; CS-200: Extracts of *C. spicatus*, medium dose group 200 mg/kg; CS-400: Extracts of *C. spicatus*, high dose group 400 mg/kg. Data are expressed as mean ± SEM (n = 10). Compared with Nor group, ^#^ *p* < 0.05, ^##^ *p* < 0.01, ^###^ *p* < 0.001; Compared with HN group, *p < 0.05, **p < 0.01, ***p < 0.001.

The XOD enzyme plays a critical role in uric acid metabolism. In this study, we found that compared to the normal group, the HN group induced by AD and PO had significantly increased (*p* < 0.001) hepatic XOD activity levels, which could promote the synthesis of more uric acid. Compared to the HN group, the low, medium, and high dose groups of mice treated with *C. spicatus* ethanol extract had significantly downregulated XOD activity levels, leading to reduced uric acid synthesis. The group treated with BEN showed no significant change in hepatic XOD activity level. The results indicate that *C. spicatus* ethanol extract could reduce the accumulation of uric acid in HN mice by inhibiting the activity of hepatic XOD and decreasing uric acid synthesis (Figure 2A). Hyperuricaemia is mainly caused by an increase in uric acid production or/and a decrease in uric acid excretion. The liver is the main site for uric acid synthesis, with PRPS as the rate-limiting enzymes. Compared to the normal group, the expression of PRPS protein in the purine *de novo* synthesis pathway was upregulated in the HN group, indicating a disorder in purine metabolism due to the abnormal activity of the key enzyme PRPS, which leads to excessive uric acid production and consequently HN. Compared to the HN group, the expression of PRPS protein in the liver was downregulated in the BEN group and the low, medium, and high dose groups of mice treated with *C. spicatus* ethanol extract. The results suggest that BEN and *C. spicatus* ethanol extract could regulate the activity of hepatic uric acid synthetic enzymes, inhibit uric acid synthesis, and thereby reduce the serum uric acid levels in the HN group (Figure 2B). Compared to the normal group, HN group exhibited upregulated expression of renal urate reabsorption transporters GLUT9 and URAT1, along with downregulated expression of urate efflux proteins OAT1 and ABCG2, suggesting enhanced urate reabsorption and diminished secretion, leading to impaired urate excretion. In contrast, the BEN group and low-dose *C. spicatus* ethanol extract group showed downregulated GLUT9 and a trend toward upregulated OAT1 and ABCG2. The medium and high-dose *C. spicatus* ethanol extract significantly downregulated GLUT9 and URAT1, along with significantly upregulating OAT1 and ABCG2. These findings indicate that *C. spicatus* ethanol extract could modulate urate transporter protein expression (by downregulating GLUT9 and URAT1, and upregulating OAT1 and ABCG2), thereby promoting urate excretion by the kidney (Figures 2D–H).

### *C. spicatus* regulated the structure of gut microbiota in HN mice

**FIGURE 3:**
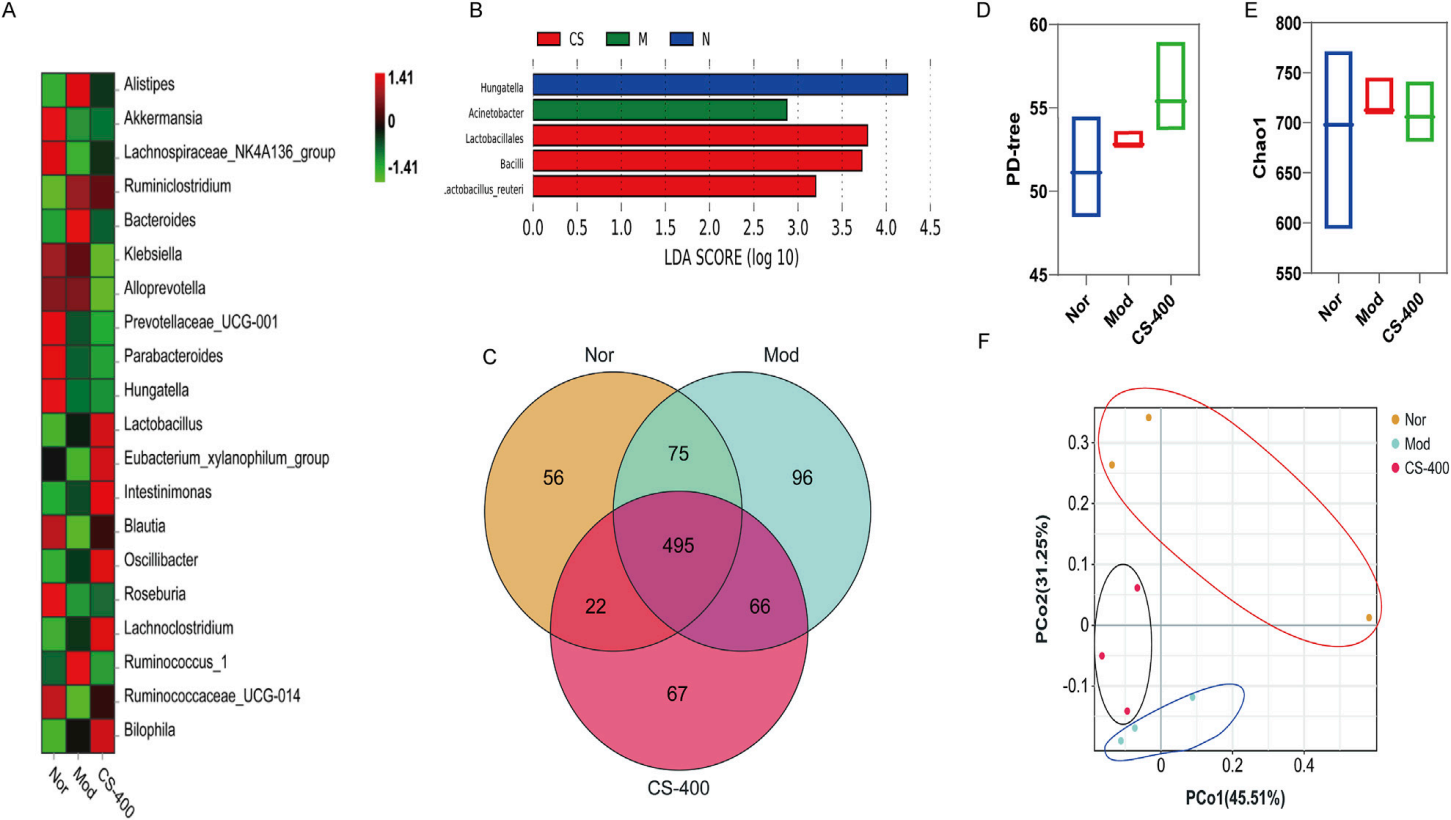
*C. spicatus* regulated the structure of gut microbiota in HN mice **(A)** Heatmap of Intestinal Bacterial Species Abundance in Mice; **(B)** LDA Score (log 10); **(C)** VENN; **(D)** PD-tree; **(E)** Chao1 index after structural damage of intestinal bacteria in mice; **(F)** PCoA; Nor: blank control group; Mod: hyperuricemia nephropathy group; CS-400: Extracts of *C. spicatus*, high dose group 400 mg/kg. Compared with Nor group, ^#^ *p* < 0.05, ^##^ *p* < 0.01, ^###^ *p* < 0.001; Compared with HN group, *p < 0.05, **p < 0.01, ***p < 0.001.

In order to study the regulatory effect of *C. spicatus* on gut microbiota, 16 S DNA was sequenced through mice feces and the results were showed that compared to the normal group, the relative abundance of *Bacteroides* and *Alistipes* in HN group increased. The relative abundance of some short-chain fatty acid producing bacteria and anti-inflammatory bacteria decreased, including *Lachnospiraceae_NK4A136_group*, *Klebsiella, Alloprevotella, Akkermansia*, and *Prevotellaceae_UCG-001*, and *Parabacteroides*. Compared to the HN group, the relative abundance of *Bacteroides* and *Alistipes* in the high-dose group of *C. spicatus* decreased (Figure 3A). To verify the bacteria that were altered by *C. spicatus* treatment, we performed high-dimensional class comparisons using linear discriminant analysis (LDA) of effect size, and sought for differences in the prevalence of bacterial communities among the three groups (Figure 3B). The results indicated that *Acinebacter* were the primary bacterial types contributing to gut microbiota dysbiosis in HN mice. In contrast, *Lactobacillales* and *Bacilli* showed relative enrichment in CS-400 group, potentially related to the CS-400 mediated alleviation the HN. Venn diagram displaying the numbers of OTUs in common between groups and unique to each group is presented (Figure 3C). However, no significant variation was observed in the α-diversity (as indicated by the PD-tree and Chao1 index) between the groups, The results indicated that CS-400 did not yield notable changes in taxa richness (Figures 3D, E). Principal coordinates analysis (PCoA) of the fecal microbiota based on Bray-Curtis distances was utilized to evaluate overall structural differences among the blank control group, HN group, and extracts of *C. spicatus* high dose group 400 mg/kg. Indicating that *C. spicatus* caused significant changes in gut microbiota composition (Figure 3F).

### *C.spicatus* regulated the abundance of associated bacteria related to uric acid metabolism and inflammation

**FIGURE 4:**
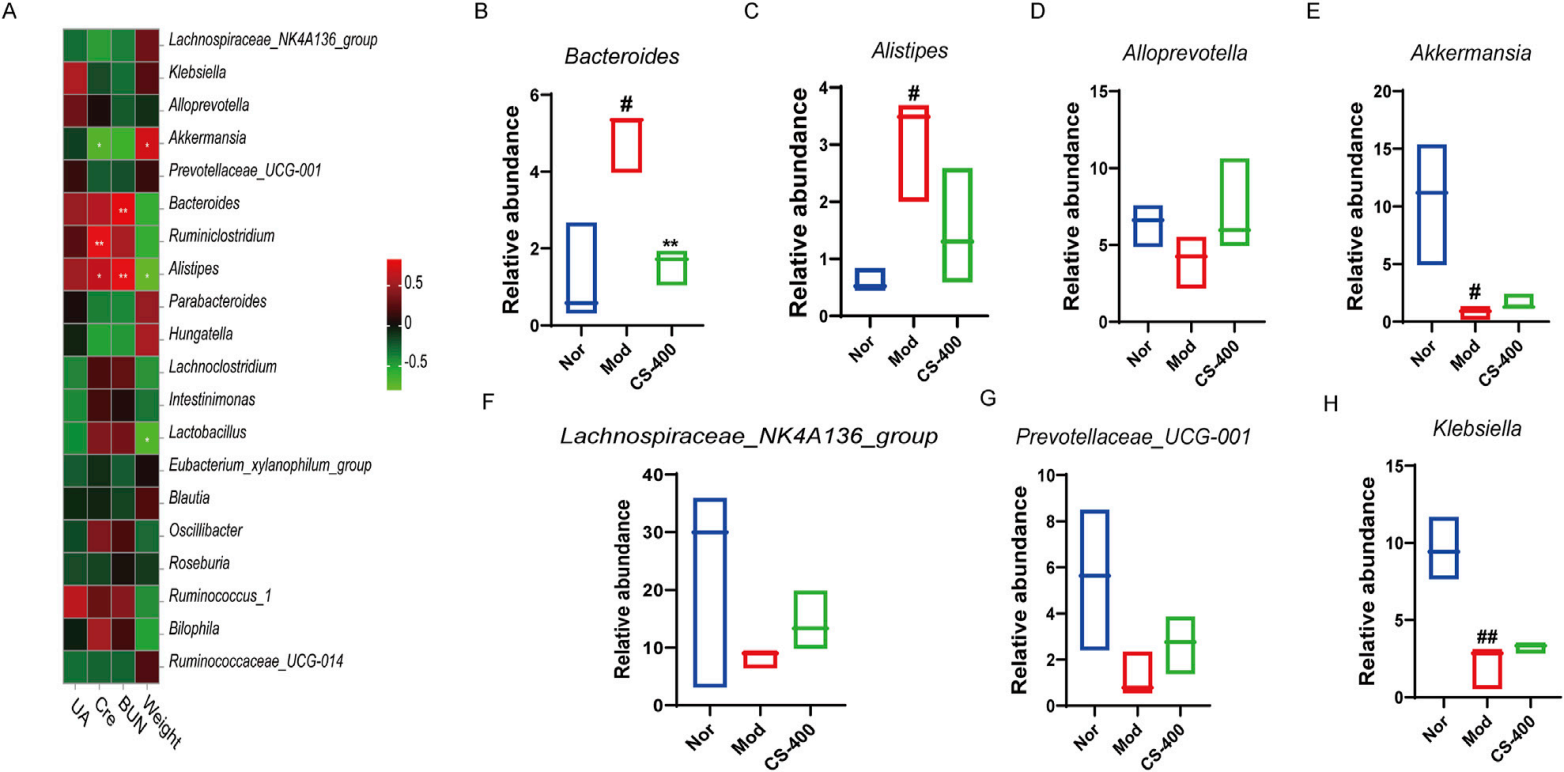
*C. spicatus* regulated the abundance of associated bacteria related to uric acid metabolism and inflammation **(A)** Correlations between environmental factors and intestinal microbes in mice were analyzed; **(B–H)** The relative abundance of 7 bacterial genera was determined. Nor: blank control group; Mod: hyperuricemia nephropathy group; CS-400: Extracts of *C. spicatus*, high dose group 400 mg/kg. Compared with Nor group, ^#^ *p* < 0.05, ^##^ *p* < 0.01, ^###^ *p* < 0.001; Compared with HN group, *p < 0.05, **p < 0.01, ***p < 0.001.

The distribution levels of *Bacteroides* and *Alistipes* in intestinal microorganisms of mice correlated positively with BUN value in serum. The distribution levels of enterobacterium *Ruminiclostridium* and *Alistipes* correlated positively with serum Cre values. The distribution level of intestinal bacterium *Akkermansia* correlated significantly negatively with serum Cre. The distribution level of intestinal bacterium *Akkermansia* was significantly negatively correlated with the serum Cre (Figure 4A). Next, we investigated the top 7 bacterial species structural changes at the genus level. Compared with the blank group, the relative abundance of *Akkermansia* and *Klebsiella* in the HN group was significantly reduced, while that of *Lachnospiraceae_NK4A136_group* and *Prevotellaceae_UCG_001* showed a decreasing trend, and the relative abundances of *Bacteroides* and *Alistipes* increased significantly. The administration of *C. spicatus* can partially reverse the dysbiosis of these bacterial taxa. The results showed that the ethanol extract of *C. spicatus* could reduce the relative abundance of *Bacteroides* and *Alistipes*, which were related to uric acid metabolism, and the increasing trend of the anti-inflammatory bacteria *Alloprevotella, Lachnospiraceae_NK4A136_group* and Short-chain fatty acid-related *Prevotellaceae_UCG_001*. Of note, *C. spicatus* administration can to some extent reverse the dysbiosis of these bacterial taxa (Figures 4B–H).

### In the pseudo-sterile mice, the effect of reducing uric acid levels by *C. spicatus* was significantly lower

**FIGURE 5:**
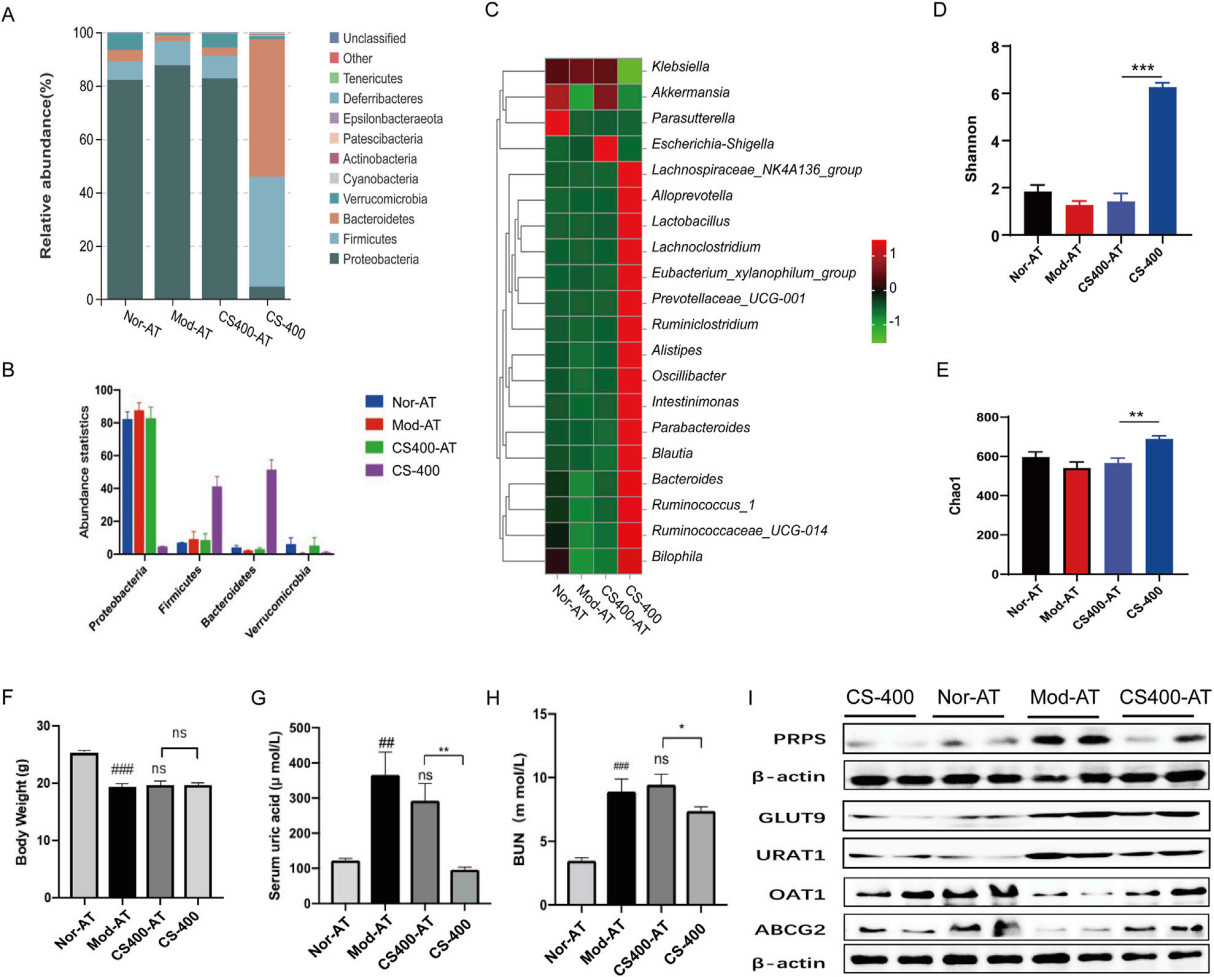
In the pseudo-sterile mice, the effect of reducing uric acid levels by *C. spicatus* was significantly lower **(A, B)** Stacked diagram of horizontal intestinal bacterial species distribution in mouse portal; **(C)** Heat map of bacterial species abundance in mouse intestine; **(D)** Shannon index; **(E)** Chao1 index; **(F)** Body Weight; **(G)** Serum Uric acid; **(H)** BUN; **(I)** Protein expression level of PRPS, GLUT9, URAT1, OAT1and ABCG2. CS-400: Extracts of *C. spicatus*, high dose group 400 mg/kg; Nor-AT: Antibiotic blank group; Mod-AT: Antibiotic hyperuricemia nephropathy group; CS400-AT: Antibiotic extracts of *C. spicatus* 400 mg/kg group. Nor-AT group compared with Mod-AT group, ^#^ *p* < 0.05, ^##^ *p* < 0.01, ^###^ *p* < 0.001; CS-400 compared with CS400-AT group, *p < 0.05, **p < 0.01, ***p < 0.001.

The previous results showed that the ethanol extract of *C. spicatus* improved gut microbiota structure, but the relationship between the therapeutic effect of the ethanol extract of *C. spicatus* on HN and gut microbiota is unclear. We administered a combination of three broad-spectrum antibiotics, ampicillin, neomycin sulfate, and vancomycin, to disrupt gut microbiota structure and simulate a sterile mouse state. This allowed us to explore the effects of gut microbiota on the uric acid lowering and renal protective effects of the ethanol extract of *C. spicatus*. The results indicated that combined antibiotic treatment significantly disrupted and eliminated the gut microbiota of mice, except for the *Proteobacteria*, with most of the gut microbiota being eliminated, resulting in a “pseudo-sterile” state (Figure 5A). In this context, the ethanol extract of *C. spicatus* had no significant regulatory effect on the gut microbiota diversity of mice (Figures 5A–E). In the pseudo-sterile state, the ethanol extract from *C. spicatus* failed to reduce body weight, serum uric acid, and blood urea nitrogen levels (Figures 5F–H). In HN mice, the synthesis of uric acid in the liver could be inhibited, but the pseudo-sterile state would weaken the inhibitory effect. Similarly, the ethanol extract from *C. spicatus* promoted renal uric acid excretion by downregulating the expression of GLUT9 and URAT1 in the kidney and upregulating the expression of uric acid secretion proteins (OAT1, ABCG2), but the pseudo-sterile state reduces its regulatory intensity (Figure 5I).

### In the pseudo-sterile mice, the effect of *C.spicatus* on pro-inflammatory cytokines expression and renal fibrosis was significantly reduced

**FIGURE 6:**
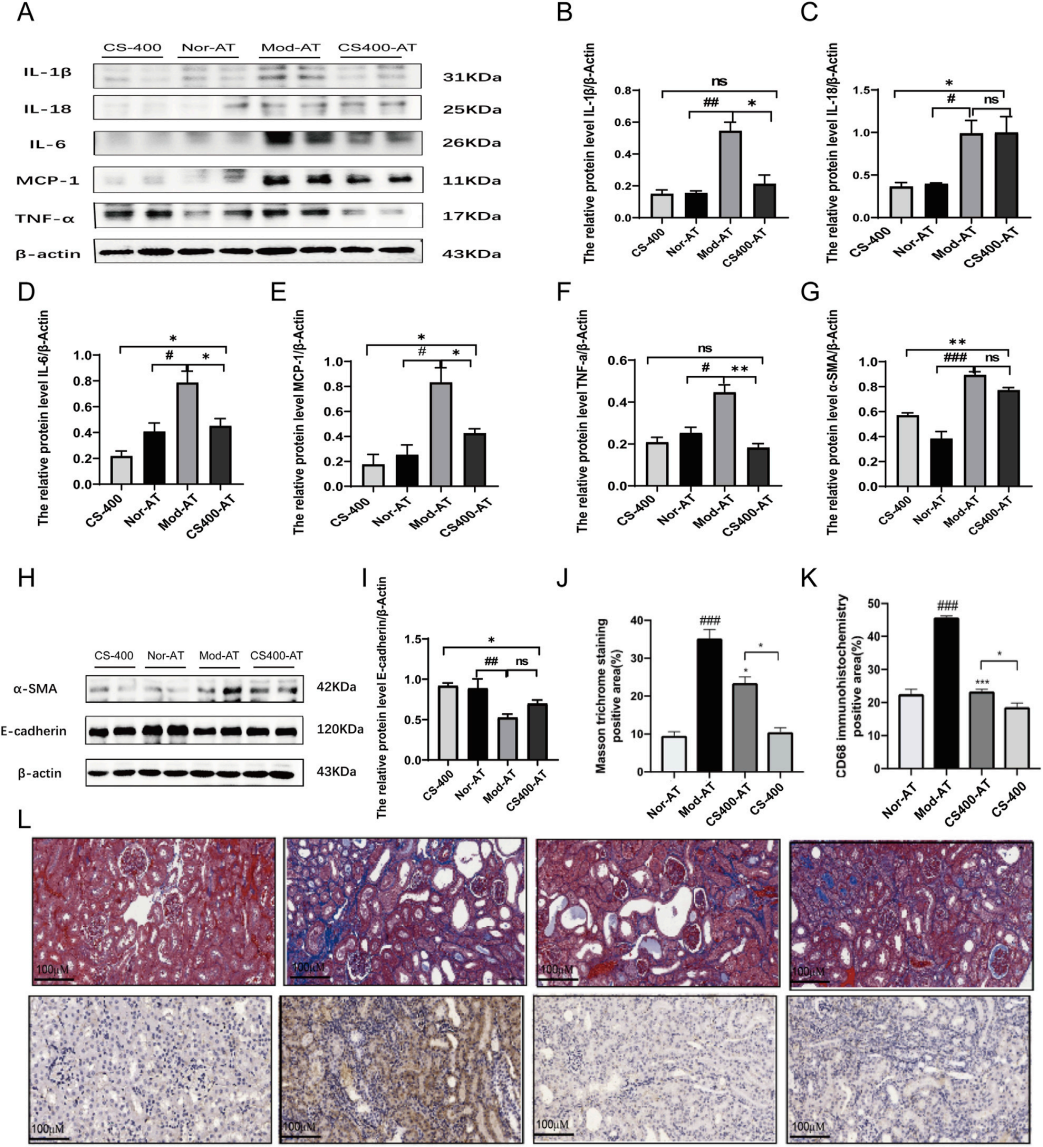
In the pseudo-sterile mice, the effect of (C) *spicatus* on pro-inflammatory cytokines expression and renal fibrosis was significantly reduced **(A)** The protein level of IL-1β, IL-18, IL-6, MCP-1 and TNF-α; **(B–F)** The relative protein level of IL-1β, IL-18, IL-6, MCP-1 and TNF-α to β-actin; **(G–I)** The protein level of α-SMA and E-cadherin and the relative protein level of α-SMA and E-cadherin to β-actin **(J–L)** Masson stained pathological sections of mouse kidney (200×) and Mouse kidney CD68 immunohistochemical staining pathological section (200×). CS-400: Extracts of *C. spicatus*, high dose group 400 mg/kg; Nor-AT: Antibiotic blank group; Mod-AT: Antibiotic hyperuricemia nephropathy group; CS400-AT: Antibiotic extracts of *C. spicatus* 400 mg/kg group. Nor-AT: Antibiotic blank group; Mod-AT: Antibiotic hyperuricemia nephropathy group; CS400-AT: Antibiotic extracts of *C. spicatus* 400 mg/kg group. Nor-AT group compared with Mod-AT group, ^#^ *p* < 0.05, ^##^ *p* < 0.01, ^###^ *p* < 0.001; CS-400 compared with CS400-AT group, *p < 0.05, **p < 0.01, ***p < 0.001.

To investigate the impact of gut microbiota on kidney inflammation and renal tubular interstitial fibrosis in HN mice, we examined the expression of relevant inflammatory factors and the state of fibrosis in the kidney. The results showed that compared to normal mice, in the pseudo-sterile mice, the expression of inflammatory factors such as IL-18, IL-6, TNF-α, and MCP-1 was significantly reduced after treatment with the ethanol extract from *C. spicatus* (Figures 6A–F). Meanwhile, compared to normal mice, the ethanol extract from *C. spicatus* in pseudo-sterile mice was still capable of reducing α-SMA protein expression in the kidney, increasing E-cadherin protein expression, but the effect of epithelial-mesenchymal transition (EMT) was reduced (Figures 6G–I). The renal pathological results were consistent with these findings, indicating that compared to normal mice, the ethanol extract from *C. spicatus* in pseudo-sterile mice could still improve the structural damage, inflammation response, macrophage accumulation, and fibrosis in the kidneys of HN mice, but the inhibitory effects were significantly reduced (Figures 6J–L). These results suggest that the gut microbiota plays a crucial role in improving kidney inflammation, fibrosis, and damage in HN mice through *C. spicatus* ethanol extract.

### The gut microbiota affected the regulation of TLR4/MYD88 and NLRP3 signaling pathways by C. spicatus

**FIGURE 7:**
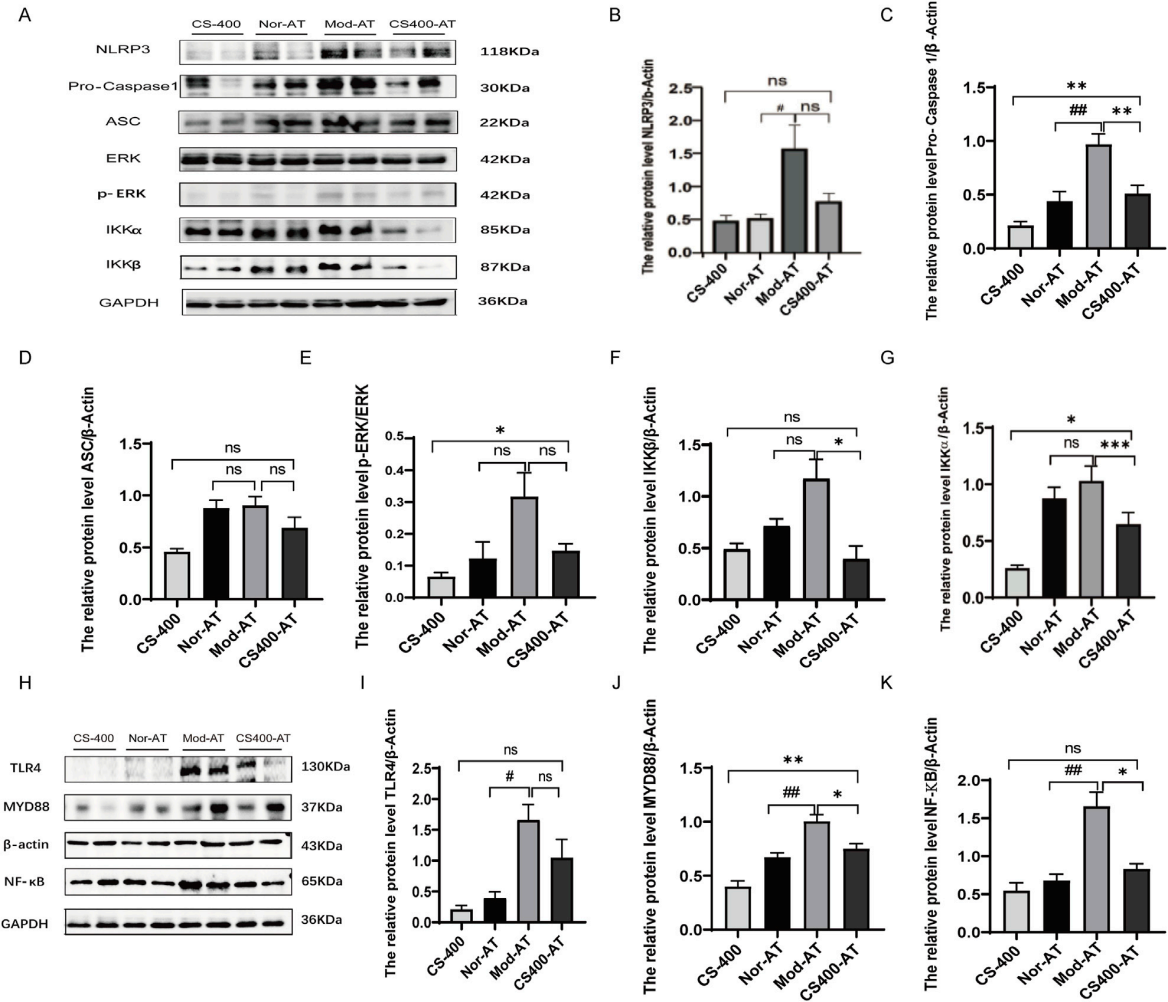
The gut microbiota affected the regulation of TLR4/MYD88 and NLRP3 signaling pathways by *C. spicatus* **(A)** Protein expression level of NLRP3 signaling pathway; **(B–G)** Protein expression level of NLRP3 signaling pathway; **(H)** The protein level of TLR4, MYD88, NF-κB; **(I–K)** The protein level of TLR4, MYD88, NF-κB. CS-400: Extracts of *C. spicatus*, high dose group 400 mg/kg; Nor-AT: Antibiotic blank group; Mod-AT: Antibiotic hyperuricemia nephropathy group; CS400-AT: Antibiotic extracts of *C. spicatus* 400 mg/kg group. Nor-AT: Antibiotic blank group; Mod-AT: Antibiotic hyperuricemia nephropathy group; CS400-AT: Antibiotic extracts of *C. spicatus* 400 mg/kg group. Nor-AT group compared with Mod-AT group, ^#^ *p* < 0.05, ^##^ *p* < 0.01, ^###^ *p* < 0.001; CS-400 compared with CS400-AT group, *p < 0.05, **p < 0.01, ***p < 0.001.

To investigate whether gut microbiota plays a role in the ameliorative effects of the ethanol extract of *C. spicatus* on HN, this study employed antibiotics to alter the gut microbiota structure in mice. The experimental results showed that while the ethanol extract of *C. spicatus* has a certain inhibitory effect on the activation of the NLRP3 and TLR4/MYD88 signaling pathways, as well as the protein expression of the NF-κB nuclear transcription factor in the kidneys of HN mice, the administration of antibiotics interfered with the inhibitory effects of the ethanol extract of *C. spicatus*. Compared to the antibiotic control group, mice in the HN group exhibited significantly upregulated protein expression levels of NLRP3, Pro-Caspase1, and Caspase1 in the kidney, associated with the NLRP3 signaling pathway. In comparison to the antibiotic-treated HN group, the group treated with 400 mg/kg of the ethanol extract of *C. spicatus* and antibiotics showed downregulated protein expression levels of Pro-Caspase1, ASC, p-ERK, IKKα, and IKKβ, as well as Caspase1, indicating suppression of NLRP3 signaling pathway activation. However, compared to the group treated with 400 mg/kg of the ethanol extract of *C. spicatus* alone, the group cotreated with antibiotics and the ethanol extract of *C. spicatus* at 400 mg/kg displayed relatively upregulated protein expression levels of Pro-Caspase1, p-ERK, and the ratio of p-ERK to ERK, as well as IKKα. The results suggest that antibiotic administration interferes with the suppressive effect of the ethanol extract of *C. spicatus* on NLRP3 signaling pathway activation (Figures 7A–G). HN group showed significant upregulation of TLR4, MYD88, and NF-κB protein expression compared to the antibiotic control group. However, treatment with the ethanol extract of *C. spicatus* and antibiotics downregulated MYD88 and NF-κB protein expression, indicating suppression of the TLR4/MYD88 and NF-κB signaling pathways. Although, compared to the group treated with t the ethanol extract of *C. spicatus* alone, the group cotreated with antibiotics and the ethanol extract of *C. spicatus* displayed relatively upregulated MYD88 protein expression. These results suggest that after antibiotic administration, the NF-κB signaling pathway in the kidneys of HN mice remains activated, but the ethanol extract of *C. spicatus* could also suppress this activation (Figures 7H–K).

## Discussion

HUA is a common metabolic disease characterized by abnormally elevated serum uric acid levels induced by excessive uric acid production and/or insufficient renal and intestinal uric acid excretion (Vareldzis et al., 2024). As the incidence of HUA rises, the prevalence of HN also increases. HN refers to kidney diseases characterized by HUA, in which uric acid accumulates in renal tissues, leading to interstitial nephritis, kidney stones, obstruction, acute or chronic renal failure (Qiao et al., 2023). HUA is considered as an independent risk factor for both acute kidney injury (AKI) and chronic kidney disease (CKD) (Shi et al., 2020). A meta-analysis involving 75,200 patients found that HUA increased the risk of AKI (Xu et al., 2017). Additionally, research has revealed that HUA can increase the risk of CKD among Chinese residents (Duan et al., 2019). For every 1 mg/dL increase in serum uric acid level, the risk of CKD increases by 49% (Saito et al., 2021). HUA is closely related to kidney disease, and the causal relationship and specific pathophysiological mechanisms have been a focal area of research (Qin et al., 2022). High uric acid deposition in renal tubules and interstitium, as well as uric acid-induced inflammation, oxidative stress, endothelial dysfunction, and fibrosis-related renal damage, constitute the direct damage caused by HUA to the kidney (Jung et al., 2020). Additionally, HUA also causes indirect kidney damage through aggravating the other risk factors of CKD (such as high blood pressure, cardiovascular disease, etc*.*) (Lin et al., 2024).

The large intestine is the primary organ responsible for excreting extracellular uric acid, accounting for approximately one-third of total uric acid excretion in the body (Maiuolo et al., 2016). When renal function is impaired, the large intestine will initiate compensatory mechanisms, eliminating more urate to maintain the body’s uric acid metabolism balance. Therefore, studying the mechanism of extracellular uric acid excretion in the large intestine may be a promising direction for the treatment of HUA and HN patients (Wen et al., 2024). Although the precise mechanism of extracellular uric acid excretion in the large intestine remains unclear, relevant research has confirmed that this process is regulated by urate transport proteins such as GLUT9 and ABCG2, and is influenced by uremic bacteria, such as *E. aerogenes*, *K. pneumoniae*, *P. aeruginosa*, etc .,(Halperin Kuhns and Woodward, 2021; Méndez-Salazar and Martínez-Nava, 2022). As early as 1968, it has been confirmed that nine bacterial species, including *E. aerogenes*, *K. pneumoniae, P*. *aeruginosa*, etc*.*, can degrade uric acid (Morgado et al., 2024). Recent studies have found that using natural medicine, such as the stem of the plant known as “Burdock” (or “Japanese burdock”), can regulate bacterial homeostasis and increase the population of beneficial bacteria (such as *Bifidobacterium*), while reducing the population of pathogenic bacteria (such as *Helicobacter pylori*), thereby lowering blood uric acid levels (Ma et al., 2023). In summary, researchers have found that the distribution of bacteria plays a significant role in maintaining uric acid balance. Therefore, for HUA and HN patients, regulating the bacterial homeostasis in the large intestine may represent a promising treatment approach that can be combined with conventional therapies.

*C. spicatus* is a perennial herb in the nepetalactone family of the Nepeta genus, with its leaves serving as a medicinal agent for the effective treatment of HUA, CKD, and urinary system stones (Chen et al., 2023; Li et al., 2024). However, the specific mechanisms remain unclear. This study demonstrated that 70% ethanol extract of *C. spicatus* inhibited uric acid synthesis by suppressing the activity of XOD and the expression of PRPS in the liver, and promoted renal uric acid excretion by regulating the expression of renal urate transporter, thereby reducing the SUA levels in HN mice. The role of the gut microbiota in the pathogenesis of HN is significant. Modulating the gut microbiota composition can improve kidney damage caused by HN. 16 S rDNA sequencing analysis results indicated that 70% ethanol extract of *C. spicatus* could reduce the abundance of *Bacteroidota* from HN mice with high uric acid accumulation, increase the relative abundance of short-chain fatty acid producers and anti-inflammatory *Lachnospiraceae_NK4A136_group*, and *Akkermansia*. These suggested that *C. spicatus* could decrease the buildup of uric acid and improve renal damage in HN mice. Therefore, we hypothesized that 70% ethanol extract of *C. spicatus* may exert its therapeutic effects on HN mice by modulating the distribution pattern of their gut microbiota.

To elucidate the role of the gut microbiota, this study utilized a combination of three broad-spectrum antibiotics (ampicillin, sulfamethoxazole, and vancomycin) to construct pseudo-sterile mice. The results indicated that after the combined antibiotic treatment, the diversity of gut microbiota in mice significantly decreased, and the bacterial community structures were similar in all groups. In the pseudo-sterile mice, 70% ethanol extract of *C. spicatus* failed to reduce the level of uric acid, alleviate renal inflammation, or improve renal fibrosis. This suggests that the gut microbiota plays a crucial role in the efficacy of *C. spicatus*. Additionally, this study revealed that the 70% ethanol extract of *C. spicatus* could significantly inhibit the activation of the NLRP3 pathway and TLR4/MYD88 pathway in the kidney of HN mice, and suppress the protein expression of nuclear transcription factor NF-κB. In pseudo-sterile mice, *C. spicatus* exhibited a significant attenuation of these signaling pathways inhibition. Based on the above analysis, we hypothesize that *C. spicatus*, through its modulation of gut microbiota, significantly reduce the uric acid level and the renal inflammatory response, and improve the renal fibrosis in HN mice.

## Conclusion

**FIGURE 8:**
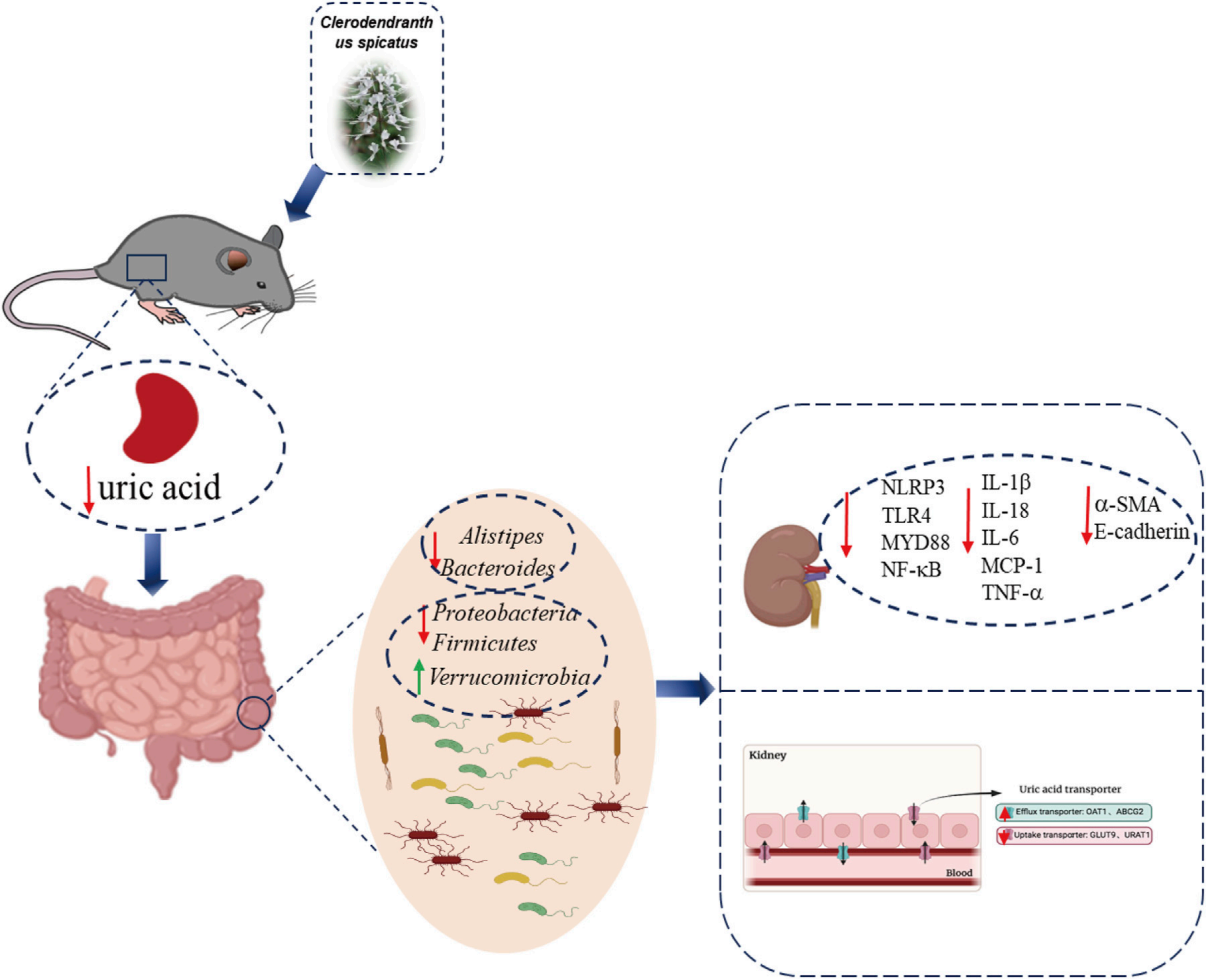
Schematic summary. *C. spicatus* could significantly reduce serum uric acid levels and improve renal function. In addition, *C. spicatus* modulated gut microbiota and decreased the relative abundance of *Bacteroides.* In pseudo-sterile HN mice, without the effect of gut microbiota, the uric acid lowering, anti-inflammatory, and renal fibrosis improving effects of *C. spicatus* were significantly reduced.

Our research indicates that *C. spicatus* regulates the gut microbiota structure in HN, reducing the relative abundance of *Bacteroides, Prevotellaceae_UCG-001* and *Alistipes, and increasing the abundance of Alloprevotella* and *Lachnospiraceae_NK4A136 _group*. *C. spicatus* alters the expression of renal uric acid transporters and key enzymes involved in liver uric acid synthesis, leading to a decrease in serum uric acid levels in HN. *C. spicatus* reduces the levels of renal inflammatory factors and alleviates renal inflammation by inhibiting the activation of NLRP3 and TLR4/MYD88 inflammatory pathways. It also improves kidney injury and fibrosis by inhibiting the process of renal epithelial mesenchymal transition. In pseudo-sterile HN mice, without the influence of gut microbiota, the uric acid lowering, anti-inflammatory, and renal fibrosis improving effects of *C. spicatus* are significantly reduced (Figure 8).

## Data Availability

The original contributions presented in the study are included in the article/Supplementary Material, further inquiries can be directed to the corresponding authors.

## Abbreviations

ABCG2, ATP-binding cassette super family G number 2; BUN, Blood urea nitrogen; Cur, Clearance of uric acid; ELISA, Enzyme linked immunosorbent assay; E-cadherin, Calcium-dependent cell adhesion molecule; GLUT9, Glucose transporter 9; H&E, Hematoxylin-eosin staining; IL-1β, Interleukin-1β; Masson, Masson-trichrome staining; MCP-1, Monocyte chemoattractant protein-1; NF-κB, Nuclear factor-kappa B; NLRP3, Nod-like receptor pyrin domain-containing protein 3; OAT1, Organic anion transporter 1; PAS, Periodic acid schiff reaction; PBS, Phosphate buffered saline; TNF-α, Tumor necrosis factor-α; UPLC, Ultra performance liquid chromatography; URAT1, Urate-anion transporter 1; α-SMA, α-Smooth muscle actin; PRPS, Phosphoribosyl pyrophosphate synthetase; XOD, Xanthine oxidase; AD, Adenine; PO, Potassium oxonate; BEN, Benzbromarone; HN, Hyperuricemic nephropathy; HUA, Hyperuricemia; AKI, Acute kidney injury.
