# Supplementary material for: Clerodendranthus spicatus [Orthosiphon aristatus (Blume) Miq.] maintains uric acid homeostasis via regulating gut microbiota and restrains renal inflammation in hyperuricemic nephropathy
Source: Front Pharmacol. 2024 Nov 25;15:1485861. doi: 10.3389/fphar.2024.1485861 (PMC11625545; doi:10.3389/fphar.2024.1485861)
Supplement: Supplementary file 1 [file DataSheet1.pdf]

Supplementary material:

Materials and methods

Sinensetin (PubChem CID: 145659) was purchased from Yuanye Biotechnology Co., Ltd., Shanghai, China. Their purity was HPLC⩾95%.

HPLC Analysis of *C. spicatus*

Sinensetin is a flavonoid isolated from the stems of *C. spicatus* and used as a quality control marker. HPLC-diode array detection analysis of sinensetin content was performed on an Agilent 1260 Infinity II system (Agilent Technology, Santa Clara, CA) using an Eclipse Plus C18 column (Agilent Technologies, USA, 4.6 × 250 mm, 5 μm) with water (A) and acetonitrile (B) as mobile phases in a gradient mode (0-18 min: 91% A, 9% B; 19-35 min: 5% A, 95% B) at ambient temperature. The content of sinensetin in EL was determined to be 0.0733%.

Result

Determination of the content of sinensetin in *C. spicatus*

Flavonoids were reported as major compounds in *C. spicatus* (Chen et al.,2020). Determination of the content of sinensetin in *C. spicatus* by HPLC method. The calibration curve showed a good linear relationship over a range of 0.01 to 0.06 mg/ml ($Y = 118381597 \times X - 8511$, $R^2 = 0.9998$). The HPLC chromatograms were shown in SFig.1, and the relative content of sinensetin in *C. spicatus* was 0.073%.

A

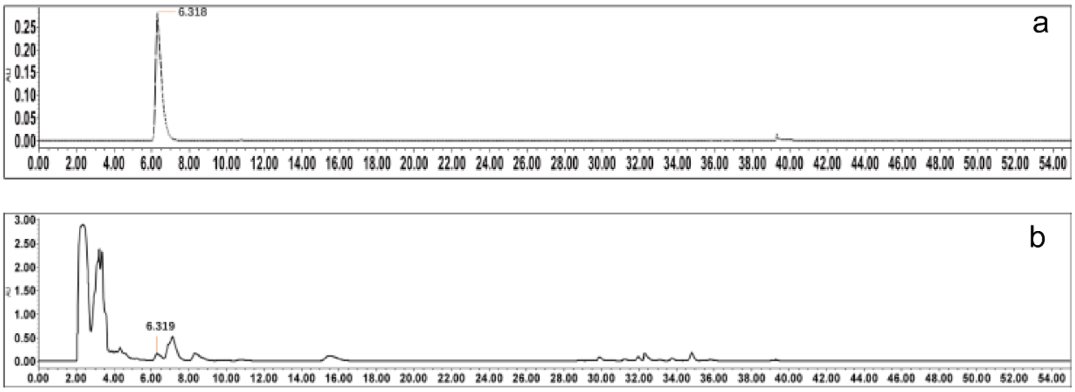

SFig.1 Determination of *C. spicatus* content (A) The relative content of sinensetin in *C. spicatus,* (a) The standard for sinensetin, (b) *C. spicatus* ethanol extract

References

Chen, W.D., Zhao, Y.L., Sun, W.J., et al., 2020. "Kidney Tea" and Its Bioactive Secondary Metabolites for Treatment of Gout. J. Agric. Food Chem.68,9131-9138.
